# Commentary: Hematuria as an early sign of multisystem inflammatory syndrome in children: A case report of a boy with multiple comorbidities and a review of the literature

**DOI:** 10.3389/fped.2022.1023525

**Published:** 2022-11-01

**Authors:** Boris Adasevic, Daniel Turudic, Danko Milosevic

**Affiliations:** ^1^ Department of Pediatrics, General Hospital Zabok and Hospital of Croatian Veterans, Bracak, Croatia; ^2^ Department of Pediatrics, University Hospital Centre Zagreb, Zagreb, Croatia; ^3^ School of Medicine, University of Zagreb, Zagreb, Croatia

**Keywords:** MIS-C, Hematuria, differential diagnosis, congenital heart disease, Child

A commentary on Hematuria as an early sign of multisystem inflammatory syndrome in children: A case report of a boy with multiple comorbidities and a review of the literature By Generalić A, Davidović M, Kos I, Vrljičak K, Lamot L. *Front Pediatr*. (2021) **9**:760070. doi: 10.3389/fped.2021.760070

## Introduction

Recently, we encountered an article that describes hematuria as the early onset of multisystem inflammatory syndrome in children (MIS-C) (1). Upon further analysis, we found some inconsistencies in the commented article, which we outlined in the following article.

## Subsections relevant to the subject

Cardiac involvement:

• Chromosome 8p23.1 deletion has been associated with severe and life-threatening congenital heart disease (CHD), requiring complex cardiac procedures and follow-ups (2). Pro-BNP levels in children with some CHD can remain elevated even after corrective surgeries (3). The type of CHD should have been presented with prior follow-ups, surgical corrections, and pro-BNP levels. Pro-BNP in CHD is primarily a marker of left ventricular systolic function, but very high levels are also an independent marker of inflammation (4–6). As increased pro-BNP is one of the markers of MIS-C cardiac inflammation and/or ventricular dysfunction, it should have trended until normalization and not presented as a single value like in the article (7).
• Common observed ECG abnormalities in MIS-C involve prolonged ECG intervals (PR, QRS complex, and QT segment), low QRS amplitude, T-wave abnormalities, or first-, second, or third-degree atrioventricular (AV) blocks (8, 9). Nodal (junctional) rhythms are uncommon in MIS-C (6.3%) and can be adequately interpreted only with a complete ECG presentation. Apart from AV blocks, they can also be a side effect of potential medications used in children with CHD (e.g., *β*-blockers, digitalis) or correction surgery for CHD (10, 11). As the ECG report before the MIS-C episode has not been presented and the described ECG report in the article is incomplete, we can only speculate on its interpretation. For instance, second- or third-degree AV block can show with normal troponin and elevated pro-BNP, just like the described case (9).
• Telemetry ECG monitoring is also recommended in patients with MIS-C with conduction delays or ectopy, which is not presented or approached in this case report (7).
• The statement that “normal values of Troponin I and creatine kinase (CK) ruled out myocarditis” is false. Troponin I and troponin T are neither specific nor sensitive markers of myocarditis (for instance, only 35% of patients with suspected viral myocarditis have elevated troponin I) (12, 13). CK used by authors is not as accurate as creatine kinase myocardial band (CK-MB) for suspected myocarditis. Cardiovascular MRI should have been at least considered, even though direct tissue examination remains the diagnostic reference standard. The indirect statement in the article that thrombocyte levels can rule out Kawasaki disease at “day 7” is also misleading, as thrombocytosis in Kawasaki disease is often seen in the second or third week of illness (1, 14).

Nephrology involvement:

• It is well known that red blood cell (RBC) morphology helps differentiate glomerular from nonglomerular hematuria. The percentage of acanthocytes >5% in urine would be beneficial, which was not done in the article (15).
• Although abnormal urinalysis was underreported in MIS-C, hematuria was scarcely found at its onset. In reported cases, it was only associated with severe acute kidney injury (AKI) or other underlying conditions (thrombotic microangiopathy (TMA), renal infarcts). No authors report spontaneous remission without treatment, which contradicts the case presented and the relevant articles (16–20). The article indirectly suggests that the hematuria was of short duration.
• Some aspects of the case report suggest a possible underlying nephrological disease as comorbidity. It is known that acute Sars-Cov-2 infection may trigger an underlying disease to manifest clinically (17, 21). IgA nephropathy manifests with periodic macrohematuria with spontaneous remission. Since we do not have a kidney biopsy, such a possibility remains unresolved.
• If the nasopharyngeal swabs are PCR-negative, Sars-Cov-2 samples could be taken from stool and urine samples. New studies isolated Sars-Cov-2 spike S1 protein from urine in 25% of patients with severe COVID-19 disease (22, 23).

Therapy:

• Despite apparent myocardial dysfunction, the patient was treated with a single 2 g/kg intravenous immune globulin (IVIG) infusion. Infusion of large volumes of IVIG (40 ml/kg) increases oncotic pressure, which may cause a liquid shift and volume overload in MIS-C patients who are already at risk due to potential capillary leak syndrome (24, 25). To cite “In patients with heart failure immunoglobulins should be administered over at least 16 h or, alternatively, the total dose should be split in two infusions 12 h apart” (26). In patients with cardiac involvement, glucocorticoids (1–2 mg/kg/day) should be “administered upfront in case of heart involvement” in conjunction with IVIG (divided/slowed infusion or with diuretics) to avoid heart overload rather than any drug alone (26, 27). Low-dose aspirin (3–5 mg/kg) is recommended in MIS-C patients with Kawasaki disease features, which is something the authors neglected or omitted from their presentation (28).

Final remarks:

1. The authors forgot or neglected to add common viral pathogens to their differential diagnosis, which can mimic or complicate MIS-C diagnosis. Pathogens that commonly include fever, exanthems, conjunctivitis, cardiomyopathy, and hematuria/hemorrhagic cystitis are adenoviruses, *Bartonella henselae*, and rarely parvovirus B19 and enteroviruses. Adenovirus can cause self-limiting hemorrhagic cystitis and myocarditis with an immediate response to IVIG therapy. Enteroviruses and Parvovirus B19 can cause glomerulonephritis, albeit rarely. Even though the symptoms match, ceftriaxone is commonly used to treat *Bartonella henselae* myocarditis (Table 1). Some authors have correctly asked the question: “Are we losing awareness of other infections due to the fear of coronavirus disease-2019 and MIS-C”? (29, 30)
2. Instead, Nino (22) in Table 1 should be Niño-Taravilla (22).

## Discussion and conclusions

The authors perhaps hastily concluded that hematuria is an early sign of MIS-C, especially in a patient with no apparent acute kidney injury (normal creatinine level). Nor is this the first published case of MIS-C-related hematuria at the time of the article's release (16, 31). Therefore, the content of the article does not correspond with the title of the article.

**Table 1:** Common (and uncommon) differential diagnostic possibilities in case report presentation.

Symptom	MIS-C	Adenovirus	Coxsackievirus	Parvovirus B19	*Bartonella henselae*
Fever	Consistently	Consistently	Consistently	Consistently	Less common
Maculopapular rash	Common	Common	Consistently	Consistently	Common
GI symptoms	Common	Less common	Common	Uncommon	Common
Macrohematuria	Very rare	Fairly common	Less common	Less common	Less common
Conjunctivitis	Common	Common	Common	Common	Less common
Pleural effusion	Common	Less common	Less common	Less common	Rare
Myocardial involvement	Common	Less common	Less common	Less common	Less common
Proteinuria	Uncommon	Uncommon	Less common	Less common	Less common

MIS-C, multisystem inflammatory syndrome in children; GI, gastrointestinal.

It is, however, a case with no apparent kidney injury but spontaneous remission of hematuria, which necessitates further diagnostic investigation. According to the presented symptoms and the clinical course of the disease, adenovirus infection appears to be the most likely cause. Subsequently, the authors should have considered the possibility of an underlying nephrological disease or bacterial/viral co-infection—with or without MIS-C.

## References

[1] Generalić A Davidović M Kos I Vrljičak K Lamot L. Hematuria as an early sign of multisystem inflammatory syndrome in children: a case report of a boy with multiple comorbidities and review of literature. Front Pediatr. (2021) 9:760070. 10.3389/fped.2021.760070 34778150 PMC8579050

[2] Wat MJ Shchelochkov OA Holder AM Breman AM Dagli A Bacino C Chromosome 8p23.1 deletions as a cause of complex congenital heart defects and diaphragmatic hernia. Am J Med Genet A. (2009) 149A(8):1661–77. 10.1002/ajmg.a.32896 19606479 PMC2765374

[3] Eindhoven JA van den Bosch AE Jansen PR Boersma E Roos-Hesselink JW. The usefulness of brain natriuretic peptide in complex congenital heart disease: a systematic review. J Am Coll Cardiol. (2012) 60(21):2140–9. 10.1016/j.jacc.2012.02.092 23021324

[4] Koch A Zink S Singer H. B-type natriuretic peptide in paediatric patients with congenital heart disease. Eur Heart J. (2006) 27(7):861–6. 10.1093/eurheartj/ehi773 16467354

[5] Sara B Monteiro JJ Carvalho P Ribeiro Carvalho C Chemba J Ferreira C Are high NT-proBNP levels more related to inflammation than to left ventricular systolic dysfunction in acute myocarditis? Eur Heart J Acute Cardiovasc Care. (2021) 10(Supplement_1):zuab020.189. 10.1093/ehjacc/zuab020.189

[6] Mannarino S Raso I Garbin M Ghidoni E Corti C Goletto S Cardiac dysfunction in multisystem inflammatory syndrome in children: an Italian single-center study. Ital J Pediatr. (2022) 48:25. 10.1186/s13052-021-01189-z 35135600 PMC8822778

[7] Alsaied T Tremoulet AH Burns JC Saidi A Dionne A Lang SM Review of cardiac involvement in multisystem inflammatory syndrome in children. Circulation. (2021) 143(1):78–88. 10.1161/CIRCULATIONAHA.120.049836 33166178

[8] Regan W O'Byrne L Stewart K Miller O Pushparajah K Theocharis P Electrocardiographic changes in children with multisystem inflammation associated with COVID-19: associated with coronavirus disease 2019. J Pediatr. (2021) 234:27–32. 10.1016/j.jpeds.2020.12.033 33358846 PMC7836928

[9] Dionne A Mah DY Son MBF Lee PY Henderson L Baker AL Atrioventricular block in children with multisystem inflammatory syndrome. Pediatrics. (2020) 146(5):e2020009704. 10.1542/peds.2020-009704 32855347

[10] Olshansky B Chung MK Pogwizd SM Goldschlager N. Chapter 3—ectopic complexes and rhythms. In: Olshansky B Chung MK Pogwizd SM Goldschlager N, editors. Arrhythmia essentials. 2nd ed. Elsevier (2017). p. 87–120. 10.1016/B978-0-323-39968-5.00003-2.

[11] Loomba RS Buelow MW Aggarwal S Arora RR Kovach J Ginde S. Arrhythmias in adults with congenital heart disease: what are risk factors for specific arrhythmias? Pacing Clin Electrophysiol. (2017) 40(4):353–61. 10.1111/pace.12983 27987225

[12] Law YM Lal AK Chen S Čiháková D Cooper LT Jr. Deshpande S Diagnosis and management of myocarditis in children: a scientific statement from the American Heart Association. Circulation. (2021) 144:e123–35. 10.1161/CIR.0000000000001001 34229446

[13] Schultz JC Hilliard AA Coope LT Rihal CS. Diagnosis and treatment of viral myocarditis. Mayo Clin Proc. (2009) 84(11):1001–9. 10.1016/S0025-6196(11)60670-8. 19880690 PMC2770911

[14] Arora K Guleria S Jindal AK Rawat A Singh S. Platelets in Kawasaki disease: is this only a numbers game or something beyond? Genes Dis. (2019) 7(1):62–6. 10.1016/j.gendis.2019.09.003 32181276 PMC7063415

[15] Bolenz C Schröppel B Eisenhardt A Schmitz-Dräger BJ Grimm MO. The investigation of hematuria. Dtsch Arztebl Int. (2018) 115(48):801–7. 10.3238/arztebl.2018.0801 30642428 PMC6365675

[16] Mahajan N Chang HT Leeman R Manalo R Glaberson WR. Case of multisystem inflammatory syndrome in children presenting as fever and abdominal pain. BMJ Case Rep. (2020) 13(9):e237306. 10.1136/bcr-2020-237306 32907870 PMC7481095

[17] Oi SSP Muniz MPR Faria IM Filho NS de Brito DJA Lages JS Multisystemic inflammatory syndrome and thrombotic microangiopathy as complications of COVID-19 in a child: a case report. Front Pediatr. (2021) 9:659069. 10.3389/fped.2021.659069 34150685 PMC8212948

[18] Sokunbi O Akinbolagbe Y Akintan P Oyeleke G Kusimo O Owowo U Clinical presentation and short-term outcomes of multisystemic inflammatory syndrome in children in Lagos, Nigeria during the COVID-19 pandemic: a case series. EClinicalMedicine. (2022) 49:101475. 10.1016/j.eclinm.2022.101475 35747195 PMC9156979

[19] Sekar N Pakkiyaretnam M Francis V. Diagnosis of multisystem inflammatory syndrome in children in a resource-limited center. Cureus. (2022) 14(2):e22254. 10.7759/cureus.22254 35340456 PMC8930290

[20] Jamee A Abotaima Z Muammar I. Multisystem inflammatory syndrome with pericardial tamponade in COVID-19: a case report. Clin Pediatr (Phila). (2022) 61(2):112–5. 10.1177/00099228211039966 34541927

[21] Fireizen Y Shahriary C Imperial ME Randhawa I Nianiaris N Ovunc B. Pediatric P-ANCA vasculitis following COVID-19. Pediatr Pulmonol. (2021) 56(10):3422–4. 10.1002/ppul.25612 34379888 PMC8441809

[22] Anjos D Fiaccadori FS Servian CDP da Fonseca SG Guilarde AO Borges MASB SARS-CoV-2 loads in urine, sera and stool specimens in association with clinical features of COVID-19 patients. J Clin Virol Plus. (2022) 2(1):100059. 10.1016/j.jcvp.2021.100059 35262032 PMC8673951

[23] George S Pal AC Gagnon J Timalsina S Singh P Vydyam P Evidence for SARS-CoV-2 spike protein in the urine of COVID-19 patients. Kidney360. (2021) 2(6):924–36. 10.34067/KID.0002172021 35373072 PMC8791366

[24] Licciardi F Baldini L Dellepiane M Covizzi C Mogni R Pruccoli G MIS-C treatment: is IVIG always necessary? Front Pediatr. (2021) 9:753123. Erratum in: Front Pediatr. (2022) **9**:826518. 10.3389/fped.2021.753123 34805048 PMC8595395

[25] Delmonaco AG Carpino A Raffaldi I Pruccoli G Garrone E Del Monte F First diagnosis of multisystem inflammatory syndrome in children (MIS-C): an analysis of PoCUS findings in the ED. Ultrasound J. (2021) 13(1):41. 10.1186/s13089-021-00243-5 34495434 PMC8424151

[26] Cattalini M Taddio A Bracaglia C Cimaz R Paolera SD Filocamo G Childhood multisystem inflammatory syndrome associated with COVID-19 (MIS-C): a diagnostic and treatment guidance from the rheumatology study group of the Italian society of pediatrics. Ital J Pediatr. (2021) 47(1):24. 10.1186/s13052-021-00980-2 33557873 PMC7868856

[27] Henderson LA Canna SW Friedman KG Gorelik M Lapidus SK Bassiri H American college of rheumatology clinical guidance for multisystem inflammatory syndrome in children associated with SARS-CoV-2 and hyperinflammation in pediatric COVID-19: version 2. Arthritis Rheumatol. (2021) 73(4):e13–29. 10.1002/art.41616 33277976 PMC8559788

[28] Algarni AS Alamri NM Khayat NZ Alabdali RA Alsubhi RS Alghamdi SH. Clinical practice guidelines in multisystem inflammatory syndrome (MIS-C) related to COVID-19: a critical review and recommendations. World J Pediatr. (2022) 18(2):83–90. 10.1007/s12519-021-00499-w 34982402 PMC8725428

[29] Ozlu SG Bayhan GI. Acute kidney injury in multisystem inflammatory syndrome in children (MIS-C). SN Compr Clin Med. (2021) 3(1):36–7. 10.1007/s42399-020-00722-1 33426470 PMC7778693

[30] Erfidan G Şimşek ÖÖ Aksay AK Üstündağ G Çamlar SA Mutlubaş F Are we losing awareness of other infections due to the fear of coronavirus disease-2019 and MIS-C? Germs. (2021) 11(4):617–24. 10.18683/germs.2021.1299 35096681 PMC8789346

[31] Grewal MK Gregory MJ Jain A Mohammad D Cashen K Ang JY Acute kidney injury in pediatric acute SARS-CoV-2 infection and multisystem inflammatory syndrome in children (MIS-C): is there a difference? Front. Pediatr. (2021) 9:692256. 10.3389/fped.2021.692256 34434905 PMC8380850

